# Cost-Utility of the eHealth Application ‘Oncokompas’, Supporting Incurably Ill Cancer Patients to Self-Manage Their Cancer-Related Symptoms: Results of a Randomized Controlled Trial

**DOI:** 10.3390/curroncol29090486

**Published:** 2022-08-27

**Authors:** Anouk S. Schuit, Karen Holtmaat, Veerle M. H. Coupé, Simone E. J. Eerenstein, Josée M. Zijlstra, Corien Eeltink, Annemarie Becker-Commissaris, Lia van Zuylen, Myra E. van Linde, C. Willemien Menke-van der Houven van Oordt, Dirkje W. Sommeijer, Nol Verbeek, Koop Bosscha, Rishi Nandoe Tewarie, Robert-Jan Sedee, Remco de Bree, Alexander de Graeff, Filip de Vos, Pim Cuijpers, Irma M. Verdonck-de Leeuw, Femke Jansen

**Affiliations:** 1 Department Clinical, Neuro and Developmental Psychology, Vrije Universiteit Amsterdam, Van der Boechorststraat 7-9, 1081 BT Amsterdam, The Netherlands; 2 Cancer Treatment and Quality of Life, Cancer Center Amsterdam, 1081 HV Amsterdam, The Netherlands; 3 Mental Health, Amsterdam Public Health, 1081 HV Amsterdam, The Netherlands; 4 Department of Epidemiology and Data Science, Amsterdam UMC, Location Vrije Universiteit Amsterdam, De Boelelaan 1117, 1081 HV Amsterdam, The Netherlands; 5 Department of Otolaryngology-Head and Neck Surgery, Amsterdam UMC, Location Vrije Universiteit Amsterdam, De Boelelaan 1117, 1081 HV Amsterdam, The Netherlands; 6 Department of Hematology, Amsterdam UMC, Location Vrije Universiteit Amsterdam, De Boelelaan 1117, 1081 HV Amsterdam, The Netherlands; 7 Department of Pulmonary Diseases, Amsterdam UMC, Location Vrije Universiteit Amsterdam, De Boelelaan 1117, 1081 HV Amsterdam, The Netherlands; 8 Department of Medical Oncology, Amsterdam UMC, LocationVrije Universiteit Amsterdam, De Boelelaan 1117, 1081 HV Amsterdam, The Netherlands; 9 Department of Internal Medicine, Amsterdam UMC, Location University of Amsterdam, Meibergdreef 9, 1012 WX Amsterdam, The Netherlands; 10 Department of Internal Medicine, Flevo Hospital, Hospitaalweg 1, 1315 RA Almere, The Netherlands; 11 Department of Oncology, St. Antonius Hospital, Soestwetering 1, 3543 AZ Utrecht, The Netherlands; 12 Department of Surgery, Jeroen Bosch Hospital, Henri Dunantstraat 1, 5223 GZ Den Bosch, The Netherlands; 13 Department of Neurosurgery, Haaglanden MC, Lijnbaan 32, 2512 VA The Hague, The Netherlands; 14 Department of Otolaryngology, Head and Neck Surgery, Haaglanden MC, Lijnbaan 32, 2512 VA The Hague, The Netherlands; 15 Department of Head and Neck Surgical Oncology, University Medical Center Utrecht, Heidelberglaan 100, 3584 CX Utrecht, The Netherlands; 16 Department of Medical Oncology, University Medical Center Utrecht, Heidelberglaan 100, 3584 CX Utrecht, The Netherlands; 17 International Institute for Psychotherapy, Babeș-Bolyai University, Str. Mihail Kogălniceanu 1, 400084 Cluj-Napoca, Romania

**Keywords:** palliative care, eHealth, cost-utility analysis, cost evaluation, incurable cancer, quality of life

## Abstract

Evidence on the cost-effectiveness of eHealth in palliative care is scarce. Oncokompas, a fully automated behavioral intervention technology, aims to support self-management in cancer patients. This study aimed to assess the cost-utility of the eHealth application Oncokompas among incurably ill cancer patients, compared to care as usual. In this randomized controlled trial, patients were randomized into the intervention group (access to Oncokompas) or the waiting-list control group (access after three months). Healthcare costs, productivity losses, and health status were measured at baseline and three months. Intervention costs were also taken into account. Non-parametric bootstrapping with 5000 replications was used to obtain 95% confidence intervals around the incremental costs and quality-adjusted life years (QALYs). A probabilistic approach was used because of the skewness of cost data. Altogether, 138 patients completed the baseline questionnaire and were randomly assigned to the intervention group (69) or the control group (69). In the base case analysis, mean total costs and mean total effects were non-significantly lower in the intervention group (−€806 and −0.01 QALYs). The probability that the intervention was more effective and less costly was 4%, whereas the probability of being less effective and less costly was 74%. Among patients with incurable cancer, Oncokompas does not impact incremental costs and seems slightly less effective in terms of QALYs, compared to care as usual. Future research on the costs of eHealth in palliative cancer care is warranted to assess the generalizability of the findings of this study.

## 1. Introduction

Incurable cancer challenges patients to deal with physical and psychological symptoms, as well as social and existential concerns [1,2,3]. eHealth solutions offer an innovative way to support cancer patients in self-managing their cancer-related symptoms. They enable patients to remain in charge of their own quality of life as long as possible by providing information and advice on how to manage side-effects of cancer and its treatment [4,5]. eHealth applications are available at any time and almost any place. Furthermore, they have the potential to improve health outcomes and reduce healthcare costs by providing resource-efficient, patient-oriented care [6].

Oncokompas was developed as a fully automatic behavioral intervention technology (BIT) to support cancer patients to adopt an active role in self-managing cancer-related symptoms [7,8]. Patients get tailored feedback and advice based on Patient Reported Outcome Measures (PROMs), and a personalized overview of supportive care services. Oncokompas is based on the stepped care principle, supporting patients to take actions to deal with their symptoms by themselves, only with professional guidance if needed. Recently, a randomized controlled trial (RCT) was conducted to determine the efficacy of Oncokompas among patients with incurable cancer, in which no significant improvements were found in patient activation (i.e., patients’ skills, knowledge, and confidence to manage their disease [9]), general self-efficacy and health-related quality of life (HRQOL) [10].

Previous research indicated that psychosocial care and eHealth interventions for cancer patients are likely to be cost-effective at different, potentially acceptable, willingness-to-pay ceilings [11,12,13]. Little evidence is available on the cost-effectiveness of eHealth interventions in palliative care and mainly focusing on telemonitoring and video conferencing [14]. To our knowledge, evidence on the cost-effectiveness or cost-utility of fully automated eHealth interventions used in palliative cancer care settings is not yet available. Economic evaluations are needed to enhance evidence-based decision-making and to create and facilitate realistic business models and payment of eHealth services [15,16]. With a cost-utility analysis (CUA), the ratio between the costs and effects of an intervention is analyzed. Effects of an intervention are often expressed using the generic measure of health gain, Quality Adjusted Life Years (QALYs) [17,18].

The aim of this study was to assess the cost-utility of the eHealth application Oncokompas among patients with incurable cancer, compared to care as usual, within the context of an RCT.

## 2. Materials and Methods

### 2.1. Study Design and Population

Detailed information on the study design can be found in previous publications [10,19]. Data on the cost-utility of Oncokompas were collected alongside an RCT to determine the efficacy of Oncokompas among adult patients (≥18 years) with incurable cancer (i.e., not having curative treatment options) [10].

Patients were recruited through healthcare professionals (e.g., medical oncologists, nurses, or nurse specialists) in six hospitals in the Netherlands (Amsterdam University Medical Centers (locations VUmc and AMC), University Medical Center Utrecht, St. Antonius Hospital, Haaglanden Medical Center, and Jeroen Bosch Hospital). Patients were included when they had a life expectancy of at least three months and when they were aware of the incurability of their cancer. Patients were excluded when they had severe cognitive impairments, poor understanding of the Dutch language, did not have access to the Internet or to an e-mail address, or when they were already familiar with Oncokompas. In addition, patients were excluded when they were too ill to participate or when participation would be too burdensome according to their healthcare professional due to the patient’s participation in other studies. All participants provided informed consent before study participation.

The study protocol was approved by the Medical Ethics Committee of VU University Medical Center (2018.224) and has been published previously [19]. This trial was registered in the Netherlands Trial Register (NTR 7494/ NL7285).

### 2.2. Randomization and Allocation

Patients who completed the baseline questionnaire were randomly allocated (1:1 ratio) to the intervention group or the control group, getting access to Oncokompas directly or after three months respectively. Randomization was performed by an independent researcher, using a computer-generated randomization scheme with a random block length of four, six, or eight. Neither the coordinating researcher nor the participants were blinded after allocation, due to the nature of the intervention.

### 2.3. Care as Usual

Patients randomized to the intervention group and the control group received care as usual, which is defined as the care provided by the oncological team or by other healthcare professionals. This includes all medical and supportive care that patients received, regardless of their study participation.

### 2.4. Intervention

Oncokompas is an eHealth application, supporting patients to adopt an active role in managing their disease. Patients navigate through Oncokompas in three steps; measure, learn, and act. First, patients are asked to fill in online Patient Reported Outcome Measures (PROMs) on the topics of their own choice, to measure the severity of their symptoms (‘Measure’). Subsequently, patients get an overview of their health status on their chosen topics, after which they get information on their symptoms and advice on how to manage their symptoms on their own (‘Learn’). In addition, patients get an overview of healthcare professionals whom they can go to when professional help is necessary (‘Act’). Oncokompas is meant as an additional form of support, not as a replacement of healthcare professionals.

### 2.5. Outcome Assessment

Outcomes measuring the efficacy of Oncokompas (i.e., patient activation, general self-efficacy, and HRQOL) were assessed at baseline (t0), after two weeks (t1), and three months after the baseline measurement (t2) [10]. Outcomes measuring the cost-utility of Oncokompas (i.e., costs and utility outcomes) were collected at t0 and t2. Costs were assessed with the Medical Consumption Questionnaire (iMCQ) and the Productivity Cost Questionnaire (iPCQ), developed by the Institute for Medical Technology Assessment (iMTA) [20,21]. The iMCQ and iPCQ measure healthcare use, help received from family and friends, and productivity losses in the previous three months, respectively. Patients’ HRQOL was measured using the EuroQol-5 Dimensions (EQ-5D-5L). The Dutch index tariff was used to transform patients’ given answers to utility scores [22].

Costs were calculated from a societal perspective and included costs of healthcare, costs for patients and their families (e.g., travelling costs, help received from family and friends), costs within other sectors (e.g., productivity losses), and intervention costs. Costs of healthcare and costs for patients and their families were calculated by multiplying the units of resource use (e.g., general practitioner (GP) visits) by the integral cost price per unit [23,24]. To calculate costs for travelling to healthcare services, the units of resource use were multiplied by the mean distance to the healthcare service times the price per kilometer. Productivity losses included losses as a result of absenteeism (absence from paid work) and presenteeism (reduced quality of the paid work performed). Absenteeism was calculated as the number of days absent from work. The friction cost method, using a friction period of 85 days, was used to calculate losses due to absenteeism [23]. Presenteeism was calculated by multiplying the days of less productivity at work by the estimated amount of lost quality of the work performed on an 11-point scale. One hour of paid work was priced at €38 (regardless of gender and age) [24]. All prices were converted to prices for 2019, using price indexes. Neither costs nor effects were discounted, due to the three months follow-up period.

Intervention costs included the costs for Oncokompas, which are estimated at €450,000 annually. These were calculated using a top-down approach and comprise the costs for ICT, product and data management, content updating, implementation, and marketing. Based on 18.000 users per year (i.e., approximately 15% of all newly diagnosed patients [25]), intervention costs per user were estimated at €25 [13].

### 2.6. Statistical Analysis

SPSS version 27 (IBM, Armonk, NY, USA) and STATA version 16 (STATA, College Station, TX, USA) were used to perform the analyses. Chi-square tests and independent t-tests were used to analyze whether randomization resulted in comparable groups of patient characteristics across study arms, as well as a Mann-Whitney U test when data were not normally distributed.

A base case intention-to-treat analysis was performed to test the cost-utility of Oncokompas compared to care as usual. In the base case analysis, all participants—who completed the first questionnaire and were allocated to a study arm—were included, imputing any missing data. Due to the differences in baseline total costs and EQ-5D score and the fact that only one follow-up measurement was available (i.e., three months after the baseline measurement), the base case analysis was corrected for baseline EQ-5D and costs.

Depending on the level of missing data (i.e., data missing on item level or questionnaire level), different methods were used for imputing missing data. When data were missing on item level (e.g., when a patient reported having visited the GP, but did not report the number of visits), assumptions were based on means per study arm (intervention or control) and time point. When data was missing on the questionnaire level, total costs or EQ-5D utility scores were imputed per time point per study arm, using multiple imputations by chained equations (predictive mean matching). Variables found to be associated with missing data (i.e., living situation), observed costs (i.e., living situation), or EQ-5D utility scores (i.e., treatment, education level, comorbidities, having children, GSE score) were included in the multiple imputation model. Ten imputed datasets were created and analyzed separately. Using Rubin’s rules (1987), the results of the 10 analyses were pooled.

The cumulative costs and the number of QALYs per patient were calculated to perform incremental cost-utility analyses. The sum of all costs measured with the iMCQ and iPCQ at t2, and the intervention costs (intervention group only), were used to calculate the total cumulative costs per patient from t0 to t2. EQ-5D utility scores measured at t2 were multiplied by the three months’ time period (time between t0 and t2) to calculate QALYs.

An incremental cost-utility ratio (ICUR) was calculated by dividing the incremental costs (mean costs in the intervention group minus mean costs in the control group) by the incremental effects (mean QALYs in the intervention group minus mean QALYs in the control group). Non-parametric bootstrapping with 5000 replications was used to obtain 95% confidence intervals around the ICUR, which were projected on a cost-utility plane. A probabilistic approach was used rather than reliance upon significance levels to describe the results due to the skewness of cost data [26].

Sensitivity analyses were performed to assess the robustness of the findings of the base case analysis, namely:

1. Not adjusting the base case analysis for baseline EQ-5D scores and baseline total costs;
2. Performing a complete case analysis among patients with complete data at all time-points;
3. Including varying intervention costs of Oncokompas (€15 and €100 per user) in the base case analysis;
4. Performing the base-case analysis from a healthcare perspective, including only healthcare costs and intervention costs;
5. Imputing data for patients who died during the study (to preclude an effect of higher mortality in the intervention group compared to the control group);
6. Excluding patients who died during the study (idem).

## 3. Results

### 3.1. Study Population

**Figure 1:**
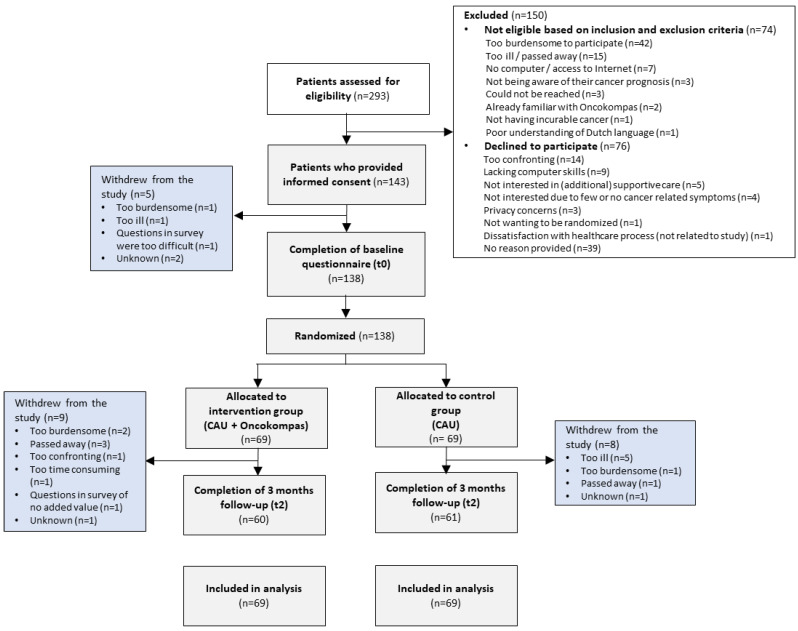
Flow diagram of the study.

Patients were recruited between December 2018 and August 2020. In total, 293 patients were screened for eligibility to participate in this study, of which 219 patients were eligible. Of these patients, 138 were willing to participate and completed the baseline questionnaire (response rate of 63%) [10]. Reasons to decline participation were: participation being too (emotionally) confronting (n = 14), lacking computer skills (n = 9), not being interested (n = 9), privacy concerns (n = 3), and other reasons (n = 5); 41 patients provided no reason for non-participation. Subsequently, patients were randomly assigned to the intervention group (n = 69) or the control group (n= 69), of which respectively 60 (87%) and 61 (88%) patients completed the follow-up questionnaire three months after the baseline measurement. No significant differences in sociodemographic and clinical characteristics were found between the intervention and control group at baseline (Table 1). Figure 1 shows a flow diagram of the study and the reasons for drop-out. Table 1 summarizes the characteristics of the 138 patients included in this study.

### 3.2. Costs and Utility Scores at Baseline and Follow-Up

Mean total costs for patients over the last three months at baseline were €4479 (SD = 4933) in the intervention group compared to €5506 (SD = 6521) in the control group. No significant differences in total costs were found between the intervention and usual care group (*p*-value = 0.30). At baseline, also no statistically significant differences were found in EQ-5D utility scores between the intervention group and control group (*p*-value = 0.35), which were respectively 0.76 (SD = 0.18) and 0.79 (SD = 0.17).

The mean costs of patients per time point per group are presented in Table 2. Complete data at t0 and t2 were available for 138 patients and 121 patients respectively. Table 3 shows the EQ-5D utility score per time point per group.

### 3.3. Cost-Utility Analyses

The results of all cost-utility analyses are presented in Table 4. In the base case analysis, mean costs and mean effects were non-significantly lower in the intervention group compared to the control group (incremental costs: −€806, 95% CI −2453 to 674, and incremental effects: −0.01 QALYs, 95% CI −0.03 to 0.001). Bootstrapping with 5000 replications was performed to assess the uncertainty surrounding the base case analysis. Of the bootstrapped cost-utility pairs, 74% fell into the south-west quadrant, indicating that the intervention was less effective and less costly. In 4% of the simulations, the intervention was more effective and less costly (south-east quadrant).

**Figure 2:**
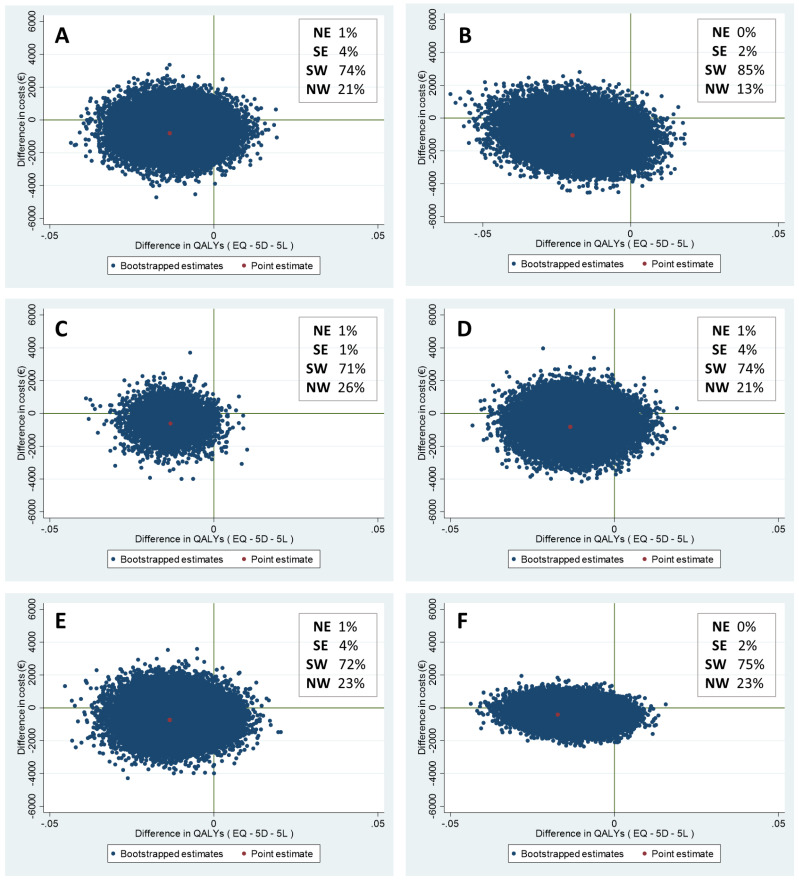

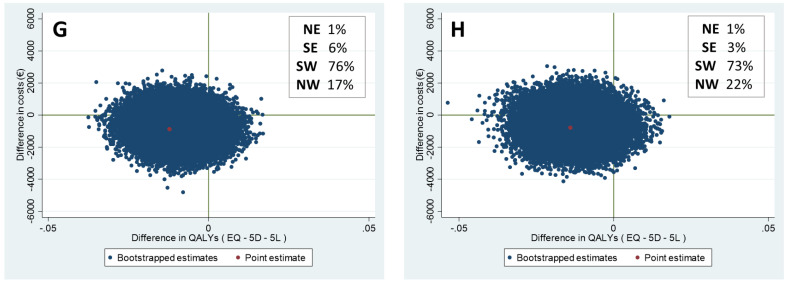
Cost-utility planes of the (**A**) base case analysis, (**B**) analysis with no correction for baseline EQ-5D score and costs, (**C**) complete case analysis, (**D**) analysis with intervention costs of €15, (**E**) analysis with intervention costs of €100, (**F**) analysis from healthcare perspective (only healthcare costs and intervention costs were taken into account), (**G**) analysis with imputed data for patients who died during the study, and (**H**) analysis excluding patients who died during the study.

To assess the robustness of the base case analysis, additional sensitivity analyses were performed (Table 4). All analyses showed non-significantly lower costs in the intervention group compared to the control group (−€990 to −€401) and non-significantly lower QALYs in the intervention group compared to the control group (−0.01 to −0.02), except for the base case analysis with no correction for baseline EQ-5D and costs and the complete case analysis (in which only patients with complete data at all time-points (i.e., t0 and t2) were included), which showed significantly lower QALYs in the intervention group compared to the control group (−0.02 and −0.01, respectively). The sensitivity analyses showed that the intervention group had a probability of 71–85% to be less effective and less costly. Figure 2 represents the cost-utility planes of all analyses.

## 4. Discussion

This study investigated the cost-utility of the eHealth self-management application Oncokompas as a behavioral intervention technology to support incurably ill cancer patients to adopt an active role in managing their disease, and improve their HRQOL. The base case analysis showed that incremental costs and incremental effects were non-significantly lower in the intervention group than in the control group (−€806 and −0.01 QALYs, respectively). These findings indicate that Oncokompas for incurably ill cancer patients does not impact incremental costs and seems slightly less effective than care as usual. The probability that the intervention is less effective and less costly was 74%.

Additional sensitivity analyses—taking into account varying intervention costs, and a healthcare perspective—confirmed the robustness of these findings, showing non-significant lower costs and effects. The sensitivity analyses taking into account only patients with complete data, and the base case analysis with no correction for baseline EQ-5D and costs, showed non-significantly lower incremental costs and significantly lower incremental effects. Two additional analyses were performed to analyze whether patients who died during the study influenced the study results: an analysis in which data was imputed for patients who died during the study (as though they were still alive) and an analysis excluding the patients who died during the study. These sensitivity analyses were performed because mortality in the intervention group was non-significantly higher in the intervention group compared to the control group (3 (5%) versus 1 (2%)). As Oncokompas is not expected to influence mortality, but a difference due to coincidence directly influences mean QALYs, these sensitivity analyses were conducted. Both analyses showed small changes in incremental costs, and the incremental QALYs showed a non-significant difference. The intervention group still had a probability of 73% to 76% that incremental QALYs and costs were lower than in the control group.

The findings of this study are in line with the findings of the parallel study on the efficacy of Oncokompas among incurably ill cancer patients (the cost outcomes were gathered alongside the trial on the efficacy of Oncokompas), which showed no improvements in patient activation, general self-efficacy, and HRQOL [10]. Earlier research indicated that palliative care services among cancer and non-cancer populations are cost-effective compared to care as usual [27,28]. However, these palliative care interventions mainly comprised hospice care, hospital-based palliative care programs, and home-based palliative care programs, and did not include eHealth interventions for use in palliative care [27,29]. To the best of our knowledge, this is the first study investigating the cost-utility of a digital health intervention in palliative cancer care. A recent study among cancer survivors treated with curative intent showed that Oncokompas was effective to improve HRQOL, while costs from a societal perspective were similar to usual cancer survivorship care [13]. In this study, the positive effects of Oncokompas on HRQOL could be merely attributed to a decrease of tumor-specific burden [30]. The content of Oncokompas for use in cancer survivorship care is developed for survivors of different cancer types specifically [8,30] (e.g., survivors of breast cancer and colorectal cancer get different content within the application). However, Oncokompas for use in palliative care is developed for incurably ill cancer patients in general, which might not be tailored enough for cancer patients to realize improvements in their HRQOL.

There has been a debate about whether the use of QALYs in palliative care is appropriate [31,32], due to changing patient values and priorities near the end-of-life and the question of whether QALYs are sensitive enough to capture the effects of a complex intervention as palliative care. QALYs enable decision makers to compare competing demands of resources and to ensure that resources are well distributed [32]. In the Dutch guideline [23], the EQ-5D-5L is the PROM of the first choice to calculate QALYs. However, the EQ-5D-5L focuses on generic symptoms and does not measure symptoms relevant for (incurable) cancer or palliative care, such as fatigue, social isolation, or spiritual symptoms (e.g., finding meaning and purpose in life) [1,2,3]. This might affect the results regarding the cost-utility in incurably ill cancer patients. It is notable that EQ-5D-5L scores in this study were relatively high among participants, which adds to the discussion on whether all aspects of HRQOL are properly measured with the EQ-5D-5L within this population. As an alternative measure, it might be interesting for future studies to use a cancer-specific, or even palliative cancer-specific utility instrument alongside the EQ-5D to investigate the cost-utility of supportive care interventions among incurably ill cancer patients [33]. In addition, it might be worthwhile to measure HRQOL from a broader perspective than just the ‘health perspective’; for example by using the Adult Social Care Outcomes Toolkit (ASCOT) [34,35].

A strength of this study is that multiple sensitivity analyses were conducted to assess the robustness of the base case analysis. Both an analysis from a societal perspective, and an analysis from a healthcare perspective were performed, including intervention costs and health care costs [36]. Another strength is the high follow-up rate, resulting in a more or less comparable percentage of participants with complete data at follow-up in both groups (87% and 88%). A limitation of this study is that the study was not powered to perform cost-utility analyses in specific sub groups, hampering the ability to, for example, conduct analyses among those who used Oncokompas as intended versus those who did not. Additionally, selection bias might have occurred, which may affect the generalizability of the study findings. Unfortunately, due to privacy regulations, no data was gathered on non-responders, hampering the possibility to compare the characteristics of responders and non-responders. Another potential limitation is that—although the number of missing data was relatively low—missing data was imputed based on assumptions (missing data on item level) or multiple imputation techniques (missing data on questionnaire level), which may not necessarily reflect reality. In addition, the results of this study might not be generalizable to other countries, since cost prices per unit and productivity losses were based on Dutch tariffs [23]. Furthermore, this study was (partly) conducted during the COVID-19 pandemic, which has affected routine palliative care and thereby the results of this study. In addition, the follow-up of this study was three months; this time frame might have been too limited to visualize the cost-saving potential of Oncokompas. Lastly, in this study, informal care costs were included to calculate the costs for patients and families. However, in this study only informal costs were included for informal caregivers’ time spent on homecare, personal care, and nursing care. When caregivers work less in a paid job due to their caregiving tasks, total informal costs made by caregivers in fact could be higher. In addition, caregiving tasks might be demanding which might result in increased costs due to presenteeism [37,38]. Future research might investigate whether the usage intensity of eHealth affects the cost-utility of eHealth interventions and to what extent total costs of eHealth interventions are affected by costs for informal caregivers.

### Study Implications

Findings of this economic evaluation of Oncokompas indicate that Oncokompas does not impact incremental costs and seems slightly less effective than care as usual among incurably ill cancer patients. Current evidence on the cost-utility of eHealth interventions is mainly focusing on telemonitoring and video conferencing; to the best of our knowledge, this study is among the first studies on cost outcomes regarding a fully automated BIT in palliative cancer care. The results of this study are limited. However, it is still possible that Oncokompas supports patients to be better informed about their symptoms and thereby being of added value to palliative cancer care. More studies in palliative cancer care are needed to put this study on the cost-utility of eHealth among incurably ill cancer patients into perspective. This is warranted since costs could be a major barrier to the implementation of eHealth interventions.

## 5. Conclusions

The fully automated behavioral intervention technology Oncokompas does not impact costs and seems slightly less effective in terms of QALYs compared to care as usual for patients with incurable cancer. This study contributes to the evidence on cost evaluations of eHealth in palliative care. However, more research on the costs of eHealth in palliative cancer care is warranted to assess the generalizability of the study findings.

## Figures and Tables

**Table 1:** Patients’ sociodemographic and clinical characteristics at baseline.

		Total Group (*n* = 138)		Control Group (*n* = 69)		Intervention Group (*n* = 69)		*p*-Value
		Number	%	Number	%	Number	%	
**Age in years**								0.29
	Mean (SD)	61.1 (12.3)	-	62.3 (11.9)	-	60.0 (12.7)	-	
	IQR	53–70.3	-	54.5–71.5	-	51.0–68.5	-	
**Gender**								1.00
	Male	74	55%	37	54%	37	54%	
	Female	64	45%	32	46%	32	46%	
**Education level**								0.61
	Low/medium/unknown	73	53%	38	55%	35	51%	
	High	65	47%	31	45%	34	49%	
**Living situation** *								0.38
	Living alone	28	20%	16	24%	12	17%	
	Living with kids/partner	109	80%	52	77%	57	83%	
**Marital status**								0.82
	Partner	115	83%	57	83%	58	84%	
	No partner	23	17%	12	17%	11	16%	
**Children**								0.69
	Yes	106	77%	54	79%	52	75%	
	No	32	23%	15	22%	17	25%	
**Employment**								0.38
	Yes	51	37%	28	41%	23	33%	
*Absent from work > 3 months*		*29*	57%	*17*	61%	*12*	52%	
	No	87	63%	41	59%	46	67%	
**Tumor type**								0.83
	Brain tumor	39	29%	22	32%	17	25%	
	Gastro-intestinal cancer	19	14%	10	15%	9	13%	
	Lung cancer	17	12%	8	12%	8	12%	
	Hematological cancer	16	12%	8	12%	8	12%	
	Head and neck cancer	16	12%	7	10%	9	13%	
	Breast cancer	15	11%	5	7%	10	15%	
	Urological cancer	10	7%	6	9%	4	6%	
	Other	4	3%	1	1%	3	6%	
	Multiple primaries ^a^	3	2%	2	3%	1	1%	
**Treatment**								0.55
	No treatment ^b^	12	9%	7	10%	5	7%	
	Single, multiple or multimodal treatment	126	91%	62	90%	64	93%	
**Comorbidities**								0.43
	None or one comorbidity	104	75%	54	78%	50	73%	
	Multiple comorbidities	34	25%	15	22%	19	28%	

^a^ Three patients were diagnosed with multiple primary tumors (one with head and neck cancer & gastro-intestinal cancer, one with lung cancer & urological cancer, and one with gastro-intestinal cancer & melanoma (other)) and are therefore shown in a separate category. ^b^ Getting no treatment also includes best supportive care and symptom management. * Missing in one patient.

**Table 2:** Mean costs per time point at t0 and t2.

			Baseline (t0)				3-Months Follow-Up (t2)			
			Intervention (*N* = 69)		Control (*N* = 69)		Intervention (*N* = 60)		Control (*N* = 61)	
		Price *	Mean	(SD)	Mean	(SD)	Mean	(SD)	Mean	(SD)
Healthcare costs			3463	(3576)	4771	(6112)	2589	(2458)	3660	(4427)
General practitioner										
	*Phone*	18	39	(38)	39	(42)	35	(42)	34	(34)
	*Home visit*	53	16	(43)	30	(68)	18	(46)	25	(59)
	*Consultation at practice*	35	53	(64)	40	(58)	40	(60)	40	(55)
Company doctor		73	28	(60)	29	(69)	30	(62)	23	(53)
Social worker		69	19	(64)	24	(72)	17	(62)	7	(24)
Physiotherapist		35	172	(293)	69	(169)	155	(203)	104	(229)
Ergotherapist		35	9	(39)	3	(18)	3	(12)	3	(16)
Dietitian		32	16	(35)	27	(62)	12	(28)	14	(44)
Speech therapist		32	2	(13)	4	(20)	1	(6)	3	(21)
Oral hygienist		27	5	(11)	6	(11)	5	(10)	4	(10)
Psychologist/psychiatrist **		100–131	109	(248)	58	(171)	66	(174)	90	(207)
Medical specialist										
	*General hospital*	85	25	(76)	38	(134)	37	(109)	21	(72)
	*Academic hospital*	174	754	(788)	1019	(1249)	696	(735)	796	(773)
Spiritual counsellor		137	20	(95)	24	(97)	14	(65)	22	(101)
Home-care (cleaning)		21	50	(184)	16	(131)	97	(259)	9	(70)
Personal care		53	48	(265)	11	(63)	25	(121)	126	(970)
Nursing care		78	43	(177)	392	(2346)	39	(264)	348	(1743)
Emergency care visit		277	72	(194)	100	(232)	83	(193)	59	(125)
Ambulance to hospital		550	40	(144)	56	(285)	55	(195)	36	(137)
Day treatment										
	*Hospital*	324	1226	(2108)	1493	(2482)	718	(1378)	1392	(2403)
	Care centre ***	72–327	0	(0)	0	(0)	0	(0)	0	(0)
Admission										
	*Hospital*	508	611	(1757)	1230	(3180)	322	(944)	425	(1296)
	Care center ***	179–491	0	(0)	0	(0)	0	(0)	0	(0)
Supportive care ****		From 15–67	105	(320)	51	(192)	114	(320)	50	(187)
**Costs for patients and their families**			**657**	**(1504)**	**462**	**(891)**	**780**	**(2866)**	**856**	**(2489)**
Transport and parking costs *****		0–9	71	(62)	82	(80)	57	(45)	71	(69)
Alternative treatment		65	2	(16)	12	(66)	7	(50)	30	(140)
Informal care		15	586	(1499)	379	(882)	723	(2865)	784	(2469)
**Other costs (i.e., productivity losses)**			**358**	**(1666)**	**273**	**(1143)**	**334**	**(2551)**	**291**	**(1129)**
Absenteeism paid work		38/hour	355	(1666)	272	(1143)	329	(2551)	287	(1113)
Presenteeism paid work		38/hour	4	(20)	1	(11)	5	(37)	4	(25)
**TOTAL COSTS**			**4479**	**(4933)**	**5506**	**(6521)**	**3703**	**(4495)**	**4806**	**(5525)**

Abbreviations: SD, standard deviation; n, sample size; * Reference price per unit (€); ** Psychologic or psychiatric help = psychological help at a private practice (€100), mental health service (out-patient) (€105), addiction clinic (€131), and/or psychologic help in hospital (€131); *** Care centre = residential centre (treatment: €72, admission: €179), rehabilitation centre (treatment: €327, admission: €491) and/or psychiatric institution (treatment: €180, admission: €323); **** Supportive care interventions = help with coping (€68), support groups (€ calculation based on price of specific support group), sport rehabilitation programs (€68), body image care (€15), self-help books (€ calculation based on answers of individual participants) and/or online self-help programs (calculation based on price of specific self-help program); ***** Transport = transportation and parking costs: €0.19/km + €3 parking costs per visit.

**Table 3:** Mean EQ-5D utility score per time point.

Time Point	N	Control Group Mean (SD)	Intervention Group Mean (SD)
**EQ-5D**			
Baseline (t0)	138	0.79 (0.17)	0.76 (0.18)
3 months follow-up (t2)	121	0.80 (0.18)	0.74 (0.21)

Abbreviations: SD, standard deviation; n, sample size.

**Table 4:** Results of the cost-utility analyses (i.e., base case and sensitivity analyses).

		Costs (€)	QALYs	Incremental Costs		Incremental Effects	
Group	N	Mean (SEM)	Mean (SEM)	€	95% CI	QALYs	95% CI
**Base case analysis** *				−806	[−2453 to 674]	−0.01	[−0.03 to 0.001]
- Control group	69	NA	NA				
- Intervention group	69	NA	NA				
Sensitivity analyses **							
**Base case analysis with no correction for baseline EQ-5D and costs**				−990	[−2690 to 594]	−0.02	[−0.04 to −0.001] ***
- Control group	69	4590 (689)	0.20 (0.01)				
- Intervention group	69	3600 (575)	0.17 (0.01)				
**Complete case analysis**				−611	[−2384 to 947]	−0.01	[−0.03 to −0.001] ***
- Control group	61	NA	NA				
- Intervention group	60	NA	NA				
**Analysis with differing intervention costs**							
€15				−816	[−2469 to 690]	−0.01	[−0.03 to 0.001]
- Control group	69	NA	NA				
- Intervention group	69	NA	NA				
€100				−731	[−2400 to 798]	−0.01	[−0.03 to 0.001]
- Control group	69	NA	NA				
- Intervention group	69	NA	NA				
**Analysis from healthcare perspective**				−401	[−1393 to 472]	−0.02	[−0.03 to 0.000]
- Control group	69	NA	NA				
- Intervention group	69	NA	NA				
**Analysis with imputed data for patients who died during the study**				−871	[−2489 to 565]	−0.01	[−0.03 to 0.003]
- Control group	69	NA	NA				
- Intervention group	69	NA	NA				
**Analysis excluding patients who died during the study**				−778	[−2430 to 742]	−0.01	[−0.03 to 0.001]
- Control group	68	NA	NA				
- Intervention group	66	NA	NA				

Abbreviations: N = sample size, SEM = standard error of the mean, 95% CI = 95% confidence interval; * The base case analysis is corrected for baseline EQ-5D utility score and costs; ** The sensitivity analyses were corrected for baseline EQ-5D utility score and costs (except the base case analysis with no correction for baseline EQ-5D and costs); *** Significant difference between the two groups (*p* < 0.05).

## Data Availability

The data presented in this study are available on request from the corresponding author.
